# Supplementary material for: Uncovering the Interrelation between Metabolite Profiles and Bioactivity of In Vitro- and Wild-Grown Catmint (Nepeta nuda L.)
Source: Metabolites. 2023 Oct 20;13(10):1099. doi: 10.3390/metabo13101099 (PMC10609352; doi:10.3390/metabo13101099)
Supplement: Supplementary file 1 [file metabolites-13-01099-s001.zip › metabolites-2664343-supplementary.pdf]

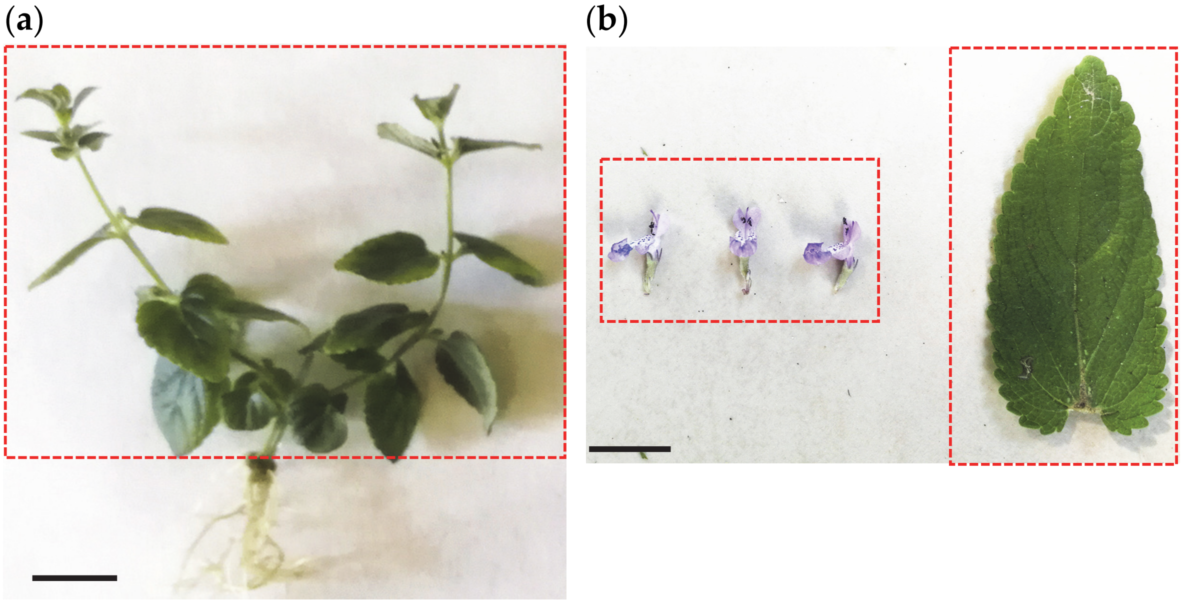

Figure S1. *Nepeta nuda* samples. Red punctuated rectangular indicates plant parts taken for analyses (**a**) Whole shoots of *in vitro* grown plants (5 weeks-old); (**b**) Individual flowers and leaves from *ex vitro* adapted *N. nuda* plants (at the phase of active blooming, at 24th of June). Scale bar: 1 cm.

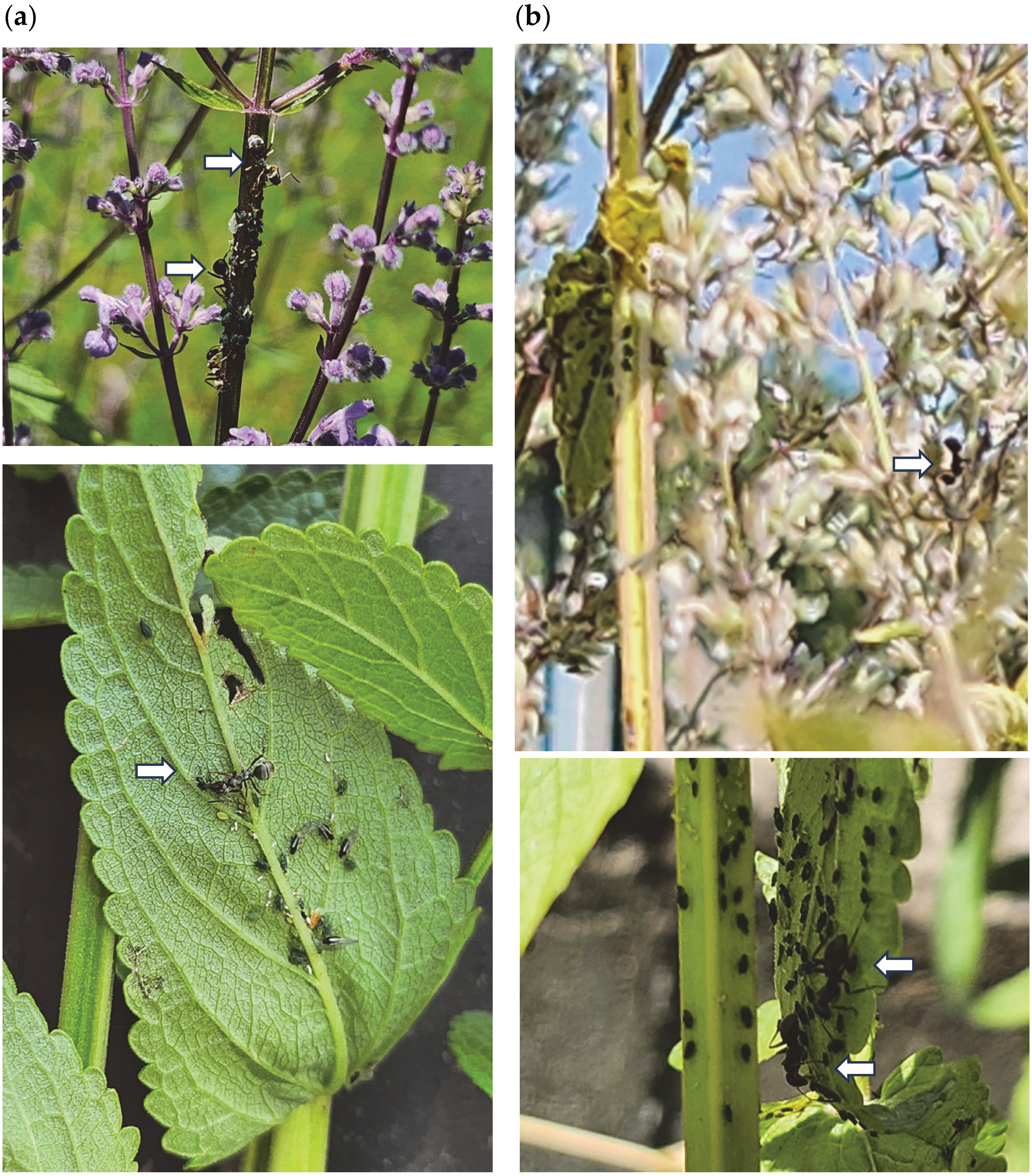

Figure S2. Ants and aphids on *N. nuda* flowers and leaves of wild-grown plants. (a) In beginning of July. (b) Three weeks later in July. Arrows point to ants.

Table S1. Pearson correlation coefficients of metabolites found in *N. nuda* polar/non-polar extracts versus biological activities: antioxidant (AO), antiviral (AV) against Human Herpes Virus 1 when simultaneously applied (AHHV1 SA) and 1-hour-post-treatment applied (AHHV1 PA), antibacterial (AB) against Gram(+) *Staphylococcus aureus* (ASa) or Gram(-) *Klebsiella pneumonia* (AKp), and anti-inflammatory; $P < 0.05$, n=3.

Metabolites		*N. nuda* biological activities						References
		AO	AHHV1SA	AHHV1PA	ASa	AKp	AI	
Total metabolites								
Phenolics		0.996		0.858				[10]
Flavonoids			0.814					[10]
Anthocyanins		1.000		0.810	0.996		0.689	AV[54]
Reducing sugars		0.994		0.868	0.980			AO[55]
Polar fraction								
P2	Malonic acid					0.906		Kp[56]
P6	Fumaric acid, 2-methyl- (Mesaconic acid)		0.837					AV[57]
P7	Citramalic acid		0.930				0.892	AV, AI[57]
P8	Malic acid	0.915			0.949	0.960		ASa[58]
P9	Erythronic acid	0.924			0.956	0.953		
P10	Tartaric acid					0.865		AKp[59]
P14	L-Valine					0.817		AKp[60]
P16	Proline				0.818	1.000		AKp[61]
P17	Glycine					0.905		AKp[62]
P18	Serine					0.837		
P28	Fructose	1.000		0.807	0.996			AV[63]
P29	Glucose	0.997			1.000	0.812		AKp[61]
P30	Mannose	0.999		0.834	0.991			AV[64]
P31	Galactose	0.994		0.867	0.980			AB, AV[65]
P32	Myo-Inositol		0.983	0.895			0.961	AV[66]
P33	Sucrose	0.919	0.879	0.976	0.878		0.919	AO[67]
P34	Trehalose, alpha,alpha'-	1.000		0.827	0.992			AV[68]
P35	Trehalose, beta,beta	0.996			1.000	0.823		AV[68]
P36	Isomaltose	0.999		0.837	0.990			AB[69]
P38	Hydroquinone	0.905	0.895	0.983	0.861		0.932	AV[70]
P39	Tyrosol	0.999		0.829	0.992			AO[71]
P40	Homovanillyl alcohol						0.934	AI[72]
P45	4-Coumaric acid		0.838					AO,AV[73]
P46	Catechollactate/Danshensu	0.986			0.997	0.863		ASa[74]
P49	Rosmarinic acid	0.996			1.000	0.823		ASa[75]
P50	Quinic acid	0.970			0.988	0.899		ASa[76]
Non-polar fraction								
NP2	Pentadecanoic acid, 14-methyl-				0.838	1.000		AB[40]
NP3	Hexadecanoic acid, 14-methyl-	0.858			0.902	0.987		AB[40]
NP5	Palmitic acid				0.812	1.000		AB[40
NP6	Linoleic acid					0.871		AB[40]
NP9	Methyl stearate	0.999			0.998			AB[40]
NP11	Eicosanoic acid (Arachidic acid)					0.945		Kp[77]
NP16	Heptadecane					0.976		AKp[78]
NP19	Pentacosane	0.990			0.999	0.850		AO, AB[79]
NP22	3-Methyltricosane	0.999			0.999			
NP23	2-Methyltetracosane	0.999		0.836	0.990			AO, AB[79]
NP24	3-Methylpentacosane	0.998			1.000	0.805		
NP27	Oleanolic acid	0.997			1.000	0.814		ASa[41]
NP28	Ursolic acid	1.000			0.998			ASa[41]

Table S2. Metabolites in polar fraction from *N. nuda*. The metabolic content was compared to in vitro plants and represented as relative values. Heat map highlights the differences in the metabolic content (maximum in red and minimum in blue). Student *t*-test was applied to determine the statistical difference relative to the in vitro variant; *$P < 0.05$, n=3.

	Polar metabolites	RT	RI	flower vs. in vitro	leaf vs. in vitro
	Organic acids				
P1	Glycolic acid	5.24	1073.1	0.2	**0.7***
P2	Malonic acid	6.63	1198.2	0.0	**−1.0***
P3	Succinic acid	7.73	1310.9	0.1	**0.4***
P4	Methylsuccinic acid	7.83	1321.9	**1.2***	**3.7***
P5	Fumaric acid	8.06	1345.6	−0.4	**1.6***
P6	Fumaric acid, 2−methyl− (Mesaconic acid)	8.52	1392.3	**2.9***	**3.9***
P7	Citramalic acid	9.18	1455.7	**1.8***	**2.2***
P8	Malic acid	9.32	1468.7	**0.9***	**−0.7***
P9	Erythronic acid	10.05	1538.0	**0.6***	**−0.3***
P10	Tartaric acid	10.96	1619.5	−0.1	**−0.9***
P11	Citric acid	13.51	1806.8	**−2.0***	**−3.2***
	Amino acids				
P12	L−Alanine	5.56	1101.9	**−0.6***	**−0.6***
P13	L−Leucine	6.16	1156.4	**−1.0***	**−1.7***
P14	L−Valine	6.75	1211.2	**−0.2***	**−1.3***
P15	L−Isoleucine	7.53	1290.5	**−0.9***	**−2.1***
P16	Proline	7.60	1298.2	**0.7***	**−2.5***
P17	Glycine	7.67	1305.0	−0.6	**−2.8***
P18	Serine	8.14	1353.1	−0.2	**−3.3***
P19	Threonine	8.37	1377.2	**−0.5***	**−2.0***
P20	Homoserine	8.96	1434.4	**−2.2***	**−3.4***
P21	L−Aspartic acid	9.66	1500.9	−0.5	**−3.1***
P22	Oxoproline	9.73	1508.3	**−4.0***	**−6.4***
P23	γ−Aminobutanoic acid (GABA)	9.81	1515.6	**−1.0***	**−3.1***
P24	L−Glutamic acid	10.80	1607.4	**−3.1***	**−4.4***
	Alcohols				
P25	Glycerol	7.31	1267.8	**0.3***	**−1.0***
P26	Galactinol	31.29	2977.0	**−2.1***	**−0.6***
	Sugar derivatives				
P27	Xylose	11.28	1643.2	**−1.9***	**−2.4***
P28	Fructose	14.36	1860.9	**3.6***	**1.0***
P29	Glucose	14.63	1878.6	**6.1***	**1.3***
P30	Mannose	14.76	1887.0	**3.8***	**1.5***
P31	Galactose	15.03	1904.1	**2.8***	**1.2***
P32	Myo−Inositol	17.90	2083.3	**0.8***	**0.9***
P33	Sucrose	26.56	2635.4	**0.7***	**0.4***
P34	Trehalose, alpha,alpha'−	27.89	2728.8	**1.5***	**0.3***
P35	Trehalose, beta,beta	28.35	2760.8	**4.4***	−0.1
P36	Isomaltose	29.57	2849.1	**4.7***	**2.2***
	Phenolic derivatives				
P37	Benzoic acid	7.14	1250.4	**−0.7***	**0.5***
P38	Hydroquinone	8.57	1397.3	**2.7***	**2.0***
P39	Tyrosol	10.30	1561.7	**2.6***	0.5
P40	Homovanillyl alcohol	11.99	1695.6	**0.8***	**1.0***
P41	Vanillic acid	12.76	1752.5	0.6	**1.9***
P42	2,5−Dihydroxybenzoic acid (Gentisic acid)	12.89	1762.2	0.5	**4.0***
P43	Shikimic acid	13.36	1796.9	−0.6	**−3.3***
P44	Syringic acid	14.76	1886.5	−1.0	**−1.4***
P45	4−Coumaric acid	15.47	1932.0	**3.3***	**4.5***
P46	Catechollactate/Danshensu	17.40	2052.7	**1.6***	−0.3
P47	Isoferulic acid	17.93	2085.2	**−2.4***	**1.5***
P48	Caffeic acid	18.67	2130.2	**1.3***	**3.7***
P49	Rosmarinic acid	37.30	3408.0	**2.9***	0.0
	Others				
P50	Quinic acid	14.11	1844.9	**2.2***	**−1.8***

Table S3. Metabolites in non-polar fraction from *N. nuda*. The metabolic content was compared to in vitro plants and represented as relative values. Heat map highlights the differences in the metabolic content (maximum in red and minimum in blue). Student *t*-test was applied to determine the statistical difference relative to the in vitro variant; **P* < 0.05, n=3.

	Non-polar metabolites	RT	RI	flower vs. in vitro	leaf vs. in vitro
	Fatty acids				
NP1	Pentadecanoic acid	13.72	1820.3	−0.6*	−0.9*
NP2	Pentadecanoic acid, 14−methyl−	14.70	1882.6	0.4	−0.8
NP3	Hexadecanoic acid, 14−methyl−	16.44	1993.9	1.1*	−3.6*
NP4	Heptadecanoic acid (Margaric acid)	16.90	2022.7	−1.0*	−0.9*
NP5	Palmitic acid	17.20	2040.6	0.2	−0.3
NP6	Linoleic acid	18.03	2091.6	−0.1	−2.2*
NP7	Linolenic acid	18.16	2099.2	−1.3*	−0.7*
NP8	Oleic acid	18.22	2103.4	−0.4*	−1.5*
NP9	Methyl stearate	18.57	2124.3	0.6	0.0
NP10	Stearic acid	20.44	2239.5	−0.1	−0.1
NP11	Eicosanoic acid (Arachidic acid)	21.82	2325.9	0.1	−0.4*
NP12	Docosanoic acid (Behenic acid)	24.96	2528.2	−0.5*	−0.7*
NP13	Tetracosanoic acid (Lignoceric acid)	27.91	2730.1	0.3*	0.6*
NP14	Methyl 2−hydroxytetracosanoate	30.33	2905.4	−0.3*	−0.5*
	Alkanes				
NP15	Hexadecane	10.68	1597.8	−0.3	−0.4*
NP16	Heptadecane	11.95	1692.5	0.0	−0.2
NP17	Octadecane	13.37	1797.7	−0.1	−0.3*
NP18	Eicosane	16.51	1998.4	−0.9*	−1.0*
NP19	Pentacosane	24.53	2500.5	1.3*	−0.1
NP20	Triacontane	31.59	2999.1	−0.4	−0.4
NP21	Dotriacontane	34.13	3199.0	−0.2	−0.2
	Branched alkanes				
NP22	3-Methyltricosane	22.55	2371.2	2.5*	0.3
NP23	2-Methyltetracosane	23.97	2462.8	2.3*	0.6
NP24	3-Methylpentacosane	25.63	2572.0	5.0*	1.0*
	Sterols				
NP25	β-Sitosterol	35.92	3317.5	−0.2*	−0.7*
NP26	α-Amyrin	36.91	3382.6	−1.2*	−0.9*
NP27	Oleanolic acid	40.44	3615.5	5.8*	0.9
NP28	Ursolic acid	41.16	3662.9	3.0*	0.6*

Table S4. Phytohormones in *N. nuda*. Heat map highlights the differences between the plant variants for each hormone. One-way ANOVA (Holm–Sidak) test was applied to determine the statistical difference between the variants (shown in different letters). In bold are highlighted the active forms of the hormones that trigger signal response by receptor binding.

**Hormones**[1]	**in vitro**	**flower**	**leaf**
Cytokinins			
Total CKs	1651.15^{a}	449.93^{c}	969.52^{b}
CK bases	**6.76^{a}**	**5.79^{a}**	**6.03^{a}**
CK ribosides	100.77^{a}	25.08^{b}	3.76^{c}
CK N-glucosides	961.48^{a}	365.10^{b}	823.90^{a}
CK O-glucosides	193.38^{a}	18.29^{c}	80.85^{b}
CK phosphates	5.22^{a}	2.06^{b}	0.56^{c}
Gibbrellins			
GA19	**4.44^{a}**	**0.62^{b}**	**1.72^{ab}**
ABAs			
ABA	**34.59^{b}**	**662.25^{a}**	**696.19^{a}**
ABA-Me	7.29^{a}	7.71^{a}	0.26^{b}
ABA-GE	582.86^{c}	3768.31^{b}	6767.03^{a}
ABA catabolites	917.54^{b}	9804.61^{a}	635.75^{b}
Jasmonates			
Total JAs	222.49^{b}	2208.81^{a}	2037.58^{a}
JA	**180.64^{c}**	**1773.33^{a}**	**363.66^{b}**
JA-Ile	16.84^{c}	400.39^{b}	1650.64^{a}
JA-Me	7.64^{b}	12.04^{a}	4.58^{b}
DiH-JA	17.37^{b}	23.04^{a}	18.70^{b}
Auxins			
IAA	**29.16^{c}**	**305.45^{a}**	**95.04^{b}**
IAA+PAA	173.82^{b}	523.36^{a}	161.77^{b}
IAA-Asp	1.25^{b}	597.69^{a}	0.67^{b}
IAA-Glu	0.41^{b}	6.74^{a}	0.07^{c}
OxIAA	23.86^{b}	29.70^{b}	65.38^{a}
IAM	1.40^{b}	3.51^{a}	0.84^{b}
I3A	164.53^{b}	2244.40^{a}	62.58^{b}
OxIAA-Glu	29.83^{a}	28.82^{a}	18.93^{a}
OxIAA-Asp	48.91^{b}	433.36^{a}	5.00^{c}
Phenolics			
SA	**146.22^{c}**	**1020.39^{a}**	**596.59^{b}**
BzA	168.21^{c}	425.55^{a}	245.63^{b}
PAAM	26.71^{b}	36.00^{a}	21.32^{b}
SinAc	2.03^{a}	0.58^{a}	0.11^{b}

[1] Metabolic forms of the hormones: **Cytokinins (CKs):** total CKs, CK bases [CK active metabolites], CK ribosides [reversible CK modification; transportation form], CK N-glucosides [inactive CK metabolites], CK O-glucosides [CK storage metabolites], CK phosphates [CK precursors]; **Gibberellin (GA)**: GA19 [active metabolite, GA precursor]; **Abscisic acid (ABA):** ABA [ABA active metabolite], ABA-Me [ABA methyl ester, metabolite], ABA-GE [ABA glucose ester, metabolite], ABA catabolites [include dihydrophaseic acid/DPA, phaseic acid/PA, 7OH-ABA, 9OH-ABA]; **Jasmonic acid (JA):** total, JA [active metabolite], JA-Ile [JA-isoleucine, active metabolite], JA-Me [JA methyl ester, metabolite], DiH-JA [dihydro-JA, metabolite]; **Auxins:** IAA [indole-3-acetic acid, active metabolite that is transported], IAA+PAA [IAA, and phenylacetic acid with auxin-like activity, but not transported], IAA-Asp [IAA-aspartate, inactive conjugate], IAA-Glu [IAA-glutamate, metabolite], OxIAA [oxo-IAA, catabolite], IAM [indole-3-acetamide, IAA precursor], I3A [indole-3-aldehyde, IAA metabolite], OxIAA-Glu [oxo-IAA-glucose ester, catabolite], OxIAA-Asp [oxo-IAA-aspartate, catabolite]; **Phenolics:** SA [salicylic acid, active metabolite], BzA [benzoic acid, SA precursor], PAAM [phenylacetamide, phenolic amide], SinAc [sinapic acid, phenolic acid].
